# Living Donor Liver Transplantation with Renoportal Anastomosis for a Patient with Congenital Absence of the Portal Vein

**DOI:** 10.1155/2012/670289

**Published:** 2012-10-03

**Authors:** Hajime Uchida, Seisuke Sakamoto, Takanobu Shigeta, Ikumi Hamano, Hiroyuki Kanazawa, Akinari Fukuda, Chiaki Karaki, Atsuko Nakazawa, Mureo Kasahara

**Affiliations:** Department of Transplant Surgery, National Center for Child Health and Development, 2-10-1 Okura, Setagaya-ku, Tokyo 157-8535, Japan

## Abstract

A congenital absence of the portal vein (CAPV) is a rare disorder that may lead to an intrapulmonary shunt. A 14-year-old male with CAPV underwent living donor liver transplantation with a left lobe graft from his father. The portal vein reconstruction was achieved with a renoportal anastomosis using an interpositional graft from the native collateral vein, because portal venous system directly drains to the left renal vein without constructing the confluence of superior mesenteric vein and splenic vein. The patient is doing well with a normal liver function and mild hypoxemia.

## 1. Introduction 

A congenital absence of the portal vein (CAPV) is a rare disorder that may lead to an intrapulmonary shunt. The complete portosystemic shunt not perfusing the liver via portal vein, defined as type I, is especially rare, but recently liver transplantation has been recognized as a curative operation for symptomatic CAPV type I patients with uncontrollable hepatic encephalopathy, pulmonary hypertension, and intrapulmonary shunt and become increasingly reported. 

## 2. Case Report

**Figure 1:**
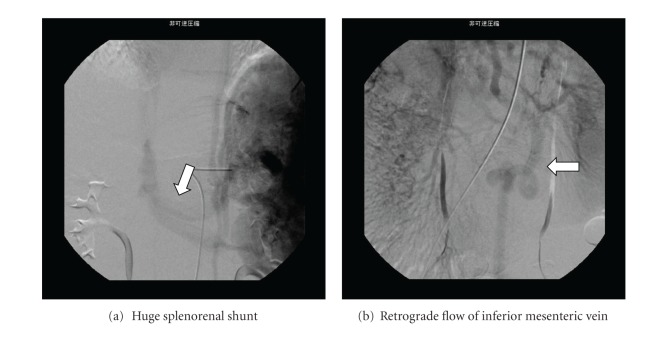
Preoperative angiography. (a) A huge splenorenal shunt (arrow) drained into the inferior vena cava without entering the hepatic hilum. (b) Retrograde flow in the dilated collateral vein (arrow) was observed.

A 14-year-old Japanese boy, who had a corrective surgery for tetralogy of Fallot at age 4, presented with hyperammonemia (serum NH3 100 *μ*mol/L), coagulopathy (PT-INR 2.19), and hypoxemia (PaO_2_ 47.2 mmHg) and was admitted to the hospital. The patient had been diagnosed with hypergalactosemia by neonatal metabolic screening. At the time of assessment on the age of 14, a laboratory evaluation showed serum NH3 115 *μ*mol/L; serum bilirubin 2.22 mg/dL; AST 30 IU/L; albumin 3.0 g/dL; total bile acid 131.3 *μ*mol/L (normal range, 10 *μ*mol/L). He presented dyspnea with prominent clubbed fingers and mild lip cyanosis and had been on long-term oxygen therapy at home. Further imaging studies revealed CAPV with a huge splenorenal shunt (Figure 1(a)), retrograde flow of a dilated collateral vein (7.5 cm in length) which could be used as a vein graft (Figure 1(b)), and severe intrapulmonary shunting (IPS: shunt ratio 56.0% by lung perfusion scintigraphy with Tc^99m^-macroaggregated albumin) without pulmonary hypertension. The portal venous system directly drains to the left renal vein without constructing the confluence of superior mesenteric vein and splenic vein. The shunt vessels were multifocal, and the ligations of these vessels were difficult to indicate. 

Though lactulose had been used to treat hyperammonemia, serum ammonia levels were still high. Due to recurrent hyperammonemia and progressive IPS despite medical treatment and protein restriction, the patient underwent living donor liver transplantation (LDLT). 

**Figure 2:**
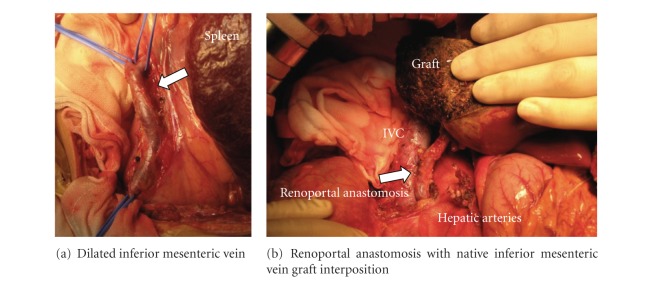
Renoportal anastomosis with the interpositional vein graft. (a) The intraoperative view shows the dilated collateral vein (arrow) procured as an interposition vein graft. (b) After the completion of the renoportal anastomosis with the vein graft (arrow).

 The donor was the patient's 39-year-old father with an incompatible blood type, and the recipient received rituximab for 4 weeks prior to LDLT and preoperative plasma exchange to reduce the complications related to ABO incompatibility [1]. The anti-A IgM/IgG titer was successfully decreased from 64/32 to 2/2 at the time of LDLT. A liver graft left sector weighing 485 g, representing 1.41% of the graft-to-recipient weight ratio, was procured. The recipient laparotomy showed CAPV, a splenorenal shunt, and retrograde flow of the collateral vein (Figure 2(a)). The recipient hepatectomy was uncomplicated. A histological examination of the 618 g explanted symmetrical native liver, which was 70.2% of the estimated standard liver volume, showed atrophic portal veins visible in the portal tracts. 

 The superior mesenteric vein and splenic vein directly drained into the left renal vein without entering the hepatic hilum. After the procurement of a 7.5 cm collateral vein for an interposition vein graft for the portal anastomosis without any noted circulatory changes, the left renal vein was divided with an autovascular stapler (PROXIMATE TX Reloadable Linear Staplers; Ethicon, USA) at its junction with the inferior vena cava (IVC). The trunk of the left renal vein, 20 mm in diameter, was anastomosed with the interposition graft with 6–0 PDS interrupted sutures. The vein graft was turned upward beside the duodenum and anastomosed directly to the graft portal vein in an end-to-end fashion with sufficient forward flow (flow volume, 921 mL/min) (Figure 2(b)). Mesenteric venous congestion did not develop. The operation lasted for 10 hours and 36 minutes, and blood loss was 1,669 mL. At the 3-year followup, the patient was doing well with a normal liver/kidney function with mild hypoxemia (shunt ratio of 35.0%).

## 3. Discussion

 CAPV is a rare disorder that may lead to hepatic encephalopathy and IPS. The portal vein derives from, embryologically, selective involution and the vitelline venous system, and its abnormality may result in CAPV. Some patients with CAPV were diagnosed at the time of neonatal screening for hypergalactosemia. LT is indicated as a curative operation for CAPV for uncontrollable hepatic encephalopathy, pulmonary hypertension, and IPS. Although pulmonary hypertension was not seen in the present patient, pathophysiology of pulmonary hypertension in CAPV is demonstrated as thromboembolic pulmonary arterial hypertension, and this state could be cured if the shunt vessel is able to close. Recently, preemptive LT for CAPV patient has become increasingly reported, because LT is the only therapeutic option to prevent regression of progressive pulmonary hypertension and IPS [2].

Securing adequate portal venous flow is crucial for successful LT. However, anastomosis between the graft portal vein and native vein may not be possible in patients with extensive portal thrombosis and/or in the absence of a proper tributary of the portal venous system [3, 4]. In the present patient, having CAPV with a significant splenorenal shunt and without confluence of superior mesenteric vein and splenic vein, the renoportal anastomosis was indicated. Renoportal anastomosis for liver allografts in cases with thrombosed PV and splenorenal/mesenteric-renal shunting has been described with excellent long-term outcome [5–10]. To the best of our knowledge, this is the first report of renoportal anastomosis in LDLT for CAPV. The renoportal anastomosis for the patient with CAPV with a significant splenorenal shunt appears to be a safe and feasible technique, which should be considered as a treatment option in LDLT.

## Abbreviations

CAPV: Congenital absence of the portal vein
HPS: Hepatopulmonary syndrome
IPS: Intrapulmonary shunting
IVC: Inferior vena cava
LDLT: Livingdonor liver transplantation
LT: Liver transplantation
PT-INR: International normalized ratio of prothrombin time
PaO_2_: Partial pressure of arterial oxygen
AST: Aspartate aminotransferase.
